# β3-adrenoreceptor blockade reduces tumor growth and increases neuronal differentiation in neuroblastoma via SK2/S1P_2_ modulation

**DOI:** 10.1038/s41388-019-0993-1

**Published:** 2019-09-02

**Authors:** Gennaro Bruno, Francesca Cencetti, Alessandro Pini, Annalisa Tondo, Daniela Cuzzubbo, Filippo Fontani, Vanessa Strinna, Anna Maria Buccoliero, Gabriella Casazza, Chiara Donati, Luca Filippi, Paola Bruni, Claudio Favre, Maura Calvani

**Affiliations:** 1 Department of Health Sciences, University of Florence, Florence, Italy; 2 Department of Paediatric Haematology-Oncology, A. Meyer University Children’s Hospital, Florence, Italy; 3 Department of Experimental and Clinical Biomedical Sciences “Mario Serio”, University of Florence, Florence, Italy; 4 Department of Experimental and Clinical Medicine, University of Florence, Florence, Italy; 5 Pathology Unit, A. Meyer University Children’s Hospital, Florence, Italy; 6 Pediatric Hematology-Oncology and Bone Marrow Transplant Unit, S. Chiara Hospital, Pisa, Italy; 7 Neonatal Intensive Care Unit, Medical Surgical Feto-Neonatal Department, A. Meyer University Children’s Hospital, Florence, Italy

**Keywords:** Lipid signalling, Paediatric cancer, Cancer stem cells

## Abstract

Neuroblastoma (NB) is the most frequently observed among extracranial pediatric solid tumors. It displays an extreme clinical heterogeneity, in particular for the presentation at diagnosis and response to treatment, often depending on cancer cell differentiation/stemness. The frequent presence of elevated hematic and urinary levels of catecholamines in patients affected by NB suggests that the dissection of adrenergic system is crucial for a better understanding of this cancer. β3-adrenoreceptor (β3-AR) is the last identified member of adrenergic receptors, involved in different tumor conditions, such as melanoma. Multiple studies have shown that the dysregulation of the bioactive lipid sphingosine 1-phosphate (S1P) metabolism and signaling is involved in many pathological diseases including cancer. However, whether S1P is crucial for NB progression and aggressiveness is still under investigation. Here we provide experimental evidence that β3-AR is expressed in NB, both human specimens and cell lines, where it is critically involved in the activation of proliferation and the regulation between stemness/differentiation, via its functional cross-talk with sphingosine kinase 2 (SK2)/S1P receptor 2 (S1P_2_) axis. The specific antagonism of β3-AR by SR59230A inhibits NB growth and tumor progression, by switching from stemness to cell differentiation both in vivo and in vitro through the specific blockade of SK2/S1P_2_ signaling.

## Introduction

Neuroblastoma (NB) is the most common extracranial solid tumor occurring in childhood. NB arises from a sympathoadrenal lineage progenitor of the neural crest during development. It is a heterogeneous malignancy with prognosis ranging from good outcome in low-risk disease to poor survival in high-risk, depending on tumor biology and clinical presentation at diagnosis. Molecular, genetic, and biological features define the different clinical behaviors, varying from complete spontaneous differentiation and regression to metastatic dissemination [1, 2]. A combination of these features is currently used to stratify patients into three groups, low-, intermediate and high-risk, according to the International Society of Pediatric Oncology Europe Neuroblastoma Group (SIOPEN) and Children’s Oncology Group (COG) [3]. An increasing number of prognostically significant genetic features, other than the well-known MYCN amplification, have been recently studied and sometimes included in developed clinical trials, in particular for relapsed/refractory disease [4]. The available therapies for treating NB are various but unfortunately not always enough effective, especially for high-risk NB. Treatment of high-risk NB remains the challenge with 5-years overall survival (OS) not yet reaching 50% related to high risk of relapse (80%) in the first 2 years from diagnosis [5]. Chemotherapy, radiotherapy, surgical resection, myeloablative therapy, and a maintenance therapy based on administration of oral retinoids, are used alone or in combination according to the specific localization and staging of the tumors.

Beta-adrenergic receptors (β-ARs) are G protein-coupled receptors, responsible for mediating vasodilation, cardiac functions, thermogenesis, and other numerous responses in health tissues. However, it is now accepted that β-ARs sustain also the pathogenesis of different cancers, from benign tumors such as the infantile hemangioma [6, 7] to several malignant tumor types including angiosarcoma [8], breast cancer [9], ovarian cancer [10], and melanoma [11, 12].

Until a few years ago, the β2-AR subtype was identified as the most involved in tumor-related pathways [13]. However, an aberrant expression of the β3-AR subtype has been recently shown in several cancers, such as leukemia, vascular tumors, colon carcinoma [14–16], and many other human cancers [17]. Moreover, recent studies have shown that the use of selective β3-AR antagonists in melanoma was effective in reducing tumor growth via direct anti-tumoral effects [18] and by affecting tumor microenvironment reactivity [19]. These results demonstrate that the β3-AR is far more widespread in tumors than previously thought, suggesting that it could play an important role in cancer biology.

Interestingly, in in vivo and in vitro NB models, non-selective β1-AR and β2-AR antagonists were able to affect tumor growth alone or in combination with chemotherapeutic agents [20, 21]. Nevertheless, whether the β3-AR subtype is expressed in NB and whether it could play a role in NB tumor biology has not been investigated so far.

Sphingosine 1-phosphate (S1P) is a powerful bioactive lipid that affects a wide variety of cellular functions such as proliferation, survival and differentiation. Many growth factors, hormones and cytokines exploit sphingosine kinase 1 (SK1) and/or SK2, the enzymes that catalyze the ATP-dependent production of S1P, to exert some of their biological effects that largely rely on binding to one or more of the five specific G protein-coupled receptors (named S1P_1–5_) [22]. There is an increasing number of literature data showing the critical role played by S1P and its metabolism in cancer [23]. While the role of SK1 in cancer has been well characterized, the contribution of SK2 has emerged only in the latest years. In fact, besides others in vitro studies, in vivo data have reported that targeting SK2 significantly reduces tumor growth in a range of human xenograft models in mice [24–26]. SK2 expression is elevated in NB cells and tissues; moreover, it has been shown that S1P, by the specific engagement of S1P_2_, induces VEGF expression, the main mediator of angiogenesis, known to be associated with NB tumor progression [27].

Our results clearly demonstrate that β3-AR is expressed in NB tumors and that its modulation strongly affects tumor growth. Moreover, we provide experimental evidence that SK2/S1P_2_ axis is responsible for β3-AR-dependent effects in NB and provide the molecular rationale to consider β3-AR/SK2/S1P_2_ as a promising therapeutic target for NB treatment.

## Results

### β3-AR is expressed in NB tumor cells

**Fig. 1:**
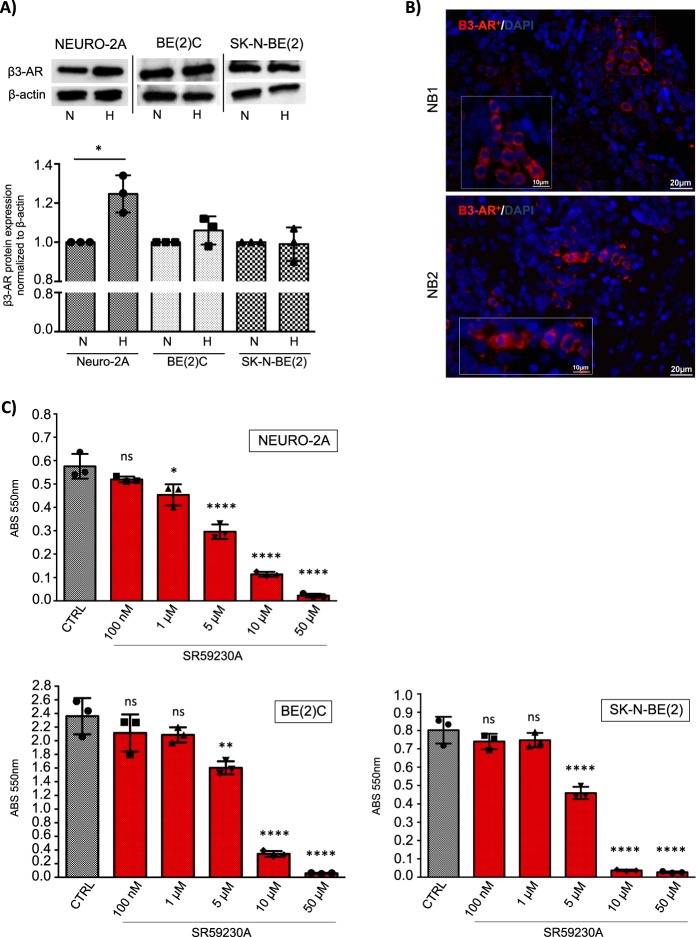
β3-AR is constitutively expressed in murine and human neuroblastoma cells and it is involved in cell survival. **a** WB and relative densitometric quantification analysis, showing expression of β3-AR protein in murine (Neuro-2A) and human (BE(2)C, SK-N-BE(2)) NB cell lines in normoxic (N) and hypoxic condition (H). Results were normalized to the expression of β-actin and reported as mean ± SD, fold change over controls (normoxic condition), set as 1. Blots are representative of three independent experiments. Significance was calculated by Unpaired *t*-test analysis with equal SD (*P < 0.05). **b** Immunofluorescence staining of β3-AR on tumor sections of two NBs (NB1, NB2) derived from human patients, showing numerous β3-AR positive cells. Images are representative of similar results obtained for *n* = 6. **c** MTT survival assay in Neuro-2A, BE(2)C and SK-N-BE(2) NB cell lines, treated with different concentration of SR59230A for 24 h. Results are reported as mean ± SD of three independent experiments performed in triplicate. Significance was calculated by one-way ANOVA analysis followed by Bonferroni’s post hoc test (ns = not significant, *P < 0.05, **P < 0.01, ****P < 0.0001)

Despite the recent findings showing that β3-AR is extensively expressed in several cancer tissues [17], the presence of this receptor in NB has not yet been investigated. To assess the expression of the β3-AR, and whether it is modulated by an hypoxic environment, we cultured three different NB cell lines, one murine (Neuro-2A) and two human (SK-N-BE(2), BE(2)C), under normoxia (21% O_2_) and hypoxia (1% O_2_) conditions for 24 h. Western blot (WB) analysis showed that β3-AR protein was expressed in all investigated NB cell lines, and that only in Neuro-2A cells, β3-AR expression was significantly increased by hypoxia (Fig. 1a). The β3-AR was found expressed also in numerous cells derived from human NB patients, as shown by immunofluorescence analysis of tumor sections (Fig. 1b). The staining procedure was validated by immunofluorescence of positive control human bronchial epithelium, previously reported to express β3-AR [28], and negative control human epidermis (Supplementary Fig. 1). The ability of several β1- and β2-blockers to exert an anti-tumor activity in NB cells has been already demonstrated [20, 21], however, the possible role played by the β3-AR in maintaining NB survival is completely unknown. To assess the effect of the β3-AR selective antagonist SR59230A on cell survival, MTT assay was employed using different concentrations of SR59230A for 24 h in the three NB cell lines. Results showed that the β3-AR antagonist was able to reduce cell viability in a dose-dependent manner (Fig. 1c), with significant effect at a concentration limit over 1 µM for Neuro-2A cells and 5 µM for SK-N-BE(2) and BE(2)C). These results suggest that the β3-AR is constitutively expressed in NB cancer cells and that it might play a crucial role in maintaining cell survival. In order to estimate off-target toxicity of SR59230A, we performed a dose-dependent cell survival MTT assay by using β3-AR antagonist in human microvascular endothelium cells HMEC-1 and human fibroblasts, IMR-90. In the above-mentioned non-tumor cell types SR59230A was not toxic at doses below 5 μM, as shown in Supplementary Fig. 2, whereas concentrations starting from 5 μM decreased cell survival in dose-dependent manner, even if at a lesser extent compared to what observed in tumor cell lines (Fig. 1c).

### β3-AR blockade modulates S1P metabolism and signaling in human NB BE(2)C cells

**Fig. 2:**
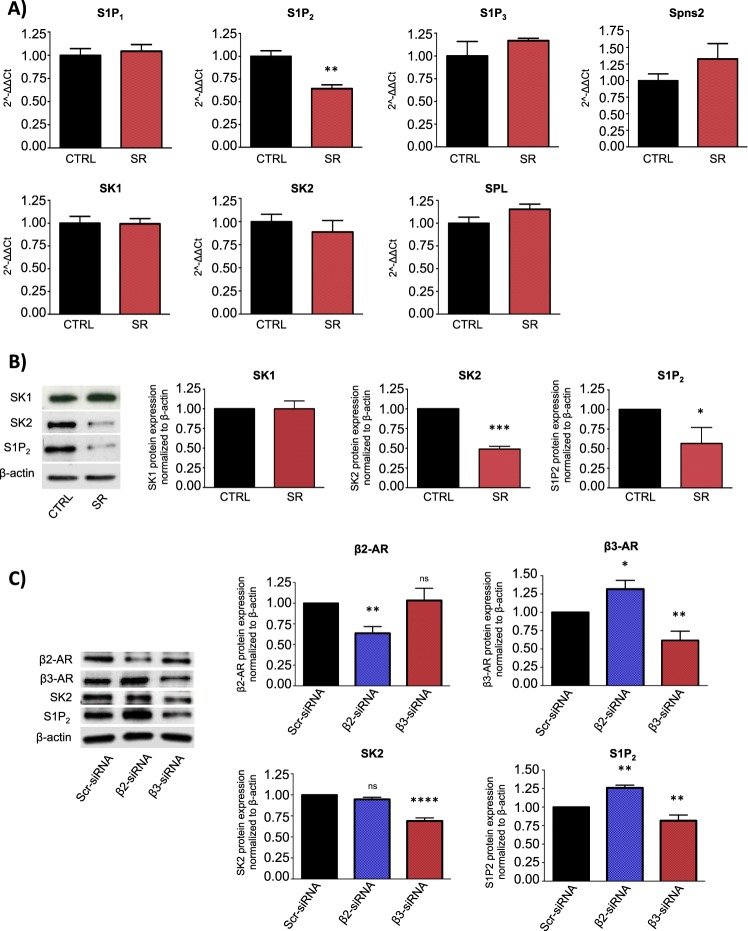
β3-AR blockade modulates S1P signaling in human neuroblastoma BE(2)C cells. **a** RT-PCR of human NB BE(2)C cell line treated with 1 μM SR59230A for 24 h. Change of mRNA expression levels of receptors (S1P_1_, S1P_2_, S1P_3_), metabolic enzymes (SK1, SK2, SPL) and transporter (Spns2) of S1P are reported as mean ± SD of three independent experiments performed in triplicate, using the 2^(-ΔΔCt) method as described in Methods section. Data were normalized to β-actin RNA expression and values of treated samples reported as fold change over control, set as 1. Significance was calculated by Unpaired *t*-test analysis with equal SD (**P < 0.01). **b** WB and relative densitometric quantification analysis, showing protein expression levels of SK1, SK2, and S1P_2_ in NB BE(2)C cell line after 24 h of 1 μM SR59230A treatment. Results were normalized to the expression of β-actin and reported as mean ± SD, fold change over control, set as 1. Blots are representative of three independent experiments. Significance was calculated by Unpaired *t*-test analysis with equal SD (*P < 0.05, ***P < 0.001). **c** WB and relative densitometric quantification analysis, showing protein expression levels of β2-AR, β3-AR, SK2 and S1P_2_ in NB BE(2)C cell line after molecular silencing of β2- and β3-AR. Results were normalized to the expression of β-actin and reported as mean ± SD, fold change over control, set as 1. Blots are representative of three independent experiments. Significance was calculated by one-way ANOVA analysis followed by Bonferroni’s post hoc test (ns = not significant, *P < 0.05, **P < 0.01, ****P < 0.0001)

Literature data showed that S1P signaling strongly affects NB progression [27, 29]. Notably, a recent paper reported a cross talk between the β3-AR and the S1P signaling in heart failure [30]. In view of this experimental data, we wondered whether these two pathways were potentially related in NB tumor biology. To address our hypothesis, we performed in vitro experiments in human BE(2)C cell line. This cell line, defined as I-type NB cells, are one of the most tumorigenic human NB cells with features of malignant neural crest stem cells, such as high self-renewal and multi-potency properties [31, 32]. Real time-PCR on BE(2)C cells treated with sublethal dose of SR59230A (1 μM) for 24 h, showed that, among the enzymes (SK1, SK2) receptors (S1P_1_, S1P_2_, S1P_3_) and S1P transporter Spinster homolog 2 (Spns2) expressed in these cells, only the S1P_2_ receptor transcript was downregulated when the β3-AR was antagonized (Fig. 2a). Nevertheless, WB analysis demonstrated that, protein levels of both S1P_2_ and SK2, were strongly reduced in BE(2)C cells treated with SR59230A for 24 h, while SK1 expression was not affected (Fig. 2b). To corroborate the pharmacological use of SR59230A as selective antagonist of β3-AR in our model, we exploited an RNA interference approach to down-regulate β2- and β3-ARs using specific siRNAs of each receptor. As shown in Fig. 2c, specific silencing of β3-AR decreased S1P_2_ and SK2, confirming the results obtained employing the β3-AR antagonist. The reduction of S1P_2_ and SK2 protein levels was not observed when β2-AR was downregulated, suggesting that the modulation of S1P_2_ and SK2 exerted by SR59230A was due to a selective β3-AR-antagonism. In addition, the selective β3-AR agonist BRL37344 significantly increased the expression of SK2 and S1P_2_ (Supplementary Fig. 3). Taken together, these results suggest the existence of a possible interplay between β3-AR and the S1P metabolism and signaling in NB cells.

### β3-AR blockade decreases cell proliferation and increases neuronal differentiation of human NB BE(2)C cells through the involvement of the S1P_2_ receptor

**Fig. 3:**
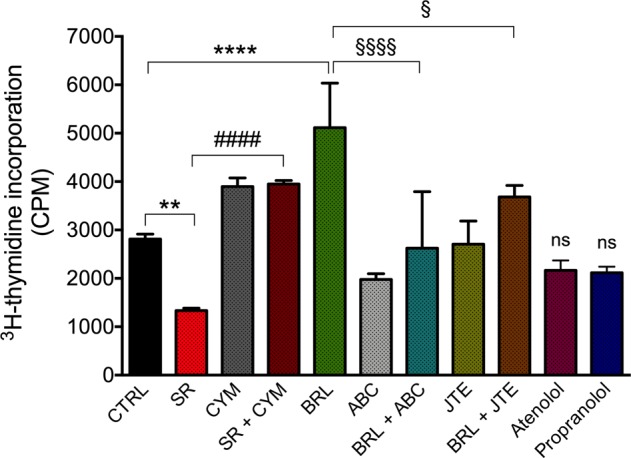
β3-AR regulates proliferation rate of human neuroblastoma BE(2)C cells through the involvement of SK2/S1P_2_ signaling. ^3^H-thymidine Proliferation Assay (1 μCi/well) of BE(2)C cell line treated with 1 μM SR59230A in presence or not of 10 μM CYM5520, 1 μM BRL37344 in presence or not of 1 μM ABC294640 and 1 μM JTE013, 1 μM Atenolol and 1 μM Propranolol for 24 h. Results are reported as mean ± SD of three independent experiments. Significance was calculated by one-way ANOVA analysis followed by Bonferroni’s post hoc test (**P < 0.01 SR vs. CTRL, ****P < 0.0001 BRL vs. CTRL; ^####^*P* < 0.0001 SR+CYM vs. SR; ^§^*P* < 0.05 BRL+JTE vs. BRL; ^§§§§^*P* < 0.0001 BRL+ABC vs. BRL)

Among others, a current therapy for treating NB cancer utilizes differentiating agents, such as retinoids [33, 34]. In melanoma cells, β3-AR has been associated with maintenance of stemness potential [19] but the molecular mechanisms responsible for this effect are still unknown. Here we investigated whether β3-AR modulation could affect the stemness/differentiation state in NB malignancy. Since the differentiation process is usually accompanied by a proliferation decrease of committed cells, first we evaluated whether β3-AR modulation could affect the proliferation of BE(2)C NB cells. A [^3^H]-thymidine incorporation proliferation assay showed that β3-AR pharmacologic blockade decreased the proliferation rate of BE(2)C NB cells, conversely, the activation of β3-AR by using the selective agonist BRL37344, elicited an increased proliferation (Fig. 3). Moreover, the anti-mitogenic action of 1 µM SR59230A was abolished in presence of the S1P_2_ agonist, CYM5520; conversely, we observed that the mitogenic action exerted by BRL37344 was reverted in presence of the SK2 inhibitor ABC294640. In agreement with the involvement of S1P_2_, the incubation with S1P_2_ antagonist, JTE013, significantly reduced BRL37344-dependent mitogenic effect. On the contrary, cell proliferation was not affected by selective β1-AR- (Atenolol) and a β1/β2-AR- (Propranolol) antagonists (Fig. 3), ruling out the possible involvement of β1/β2-AR in SR59230A-dependent anti-proliferative action.

**Fig. 4:**
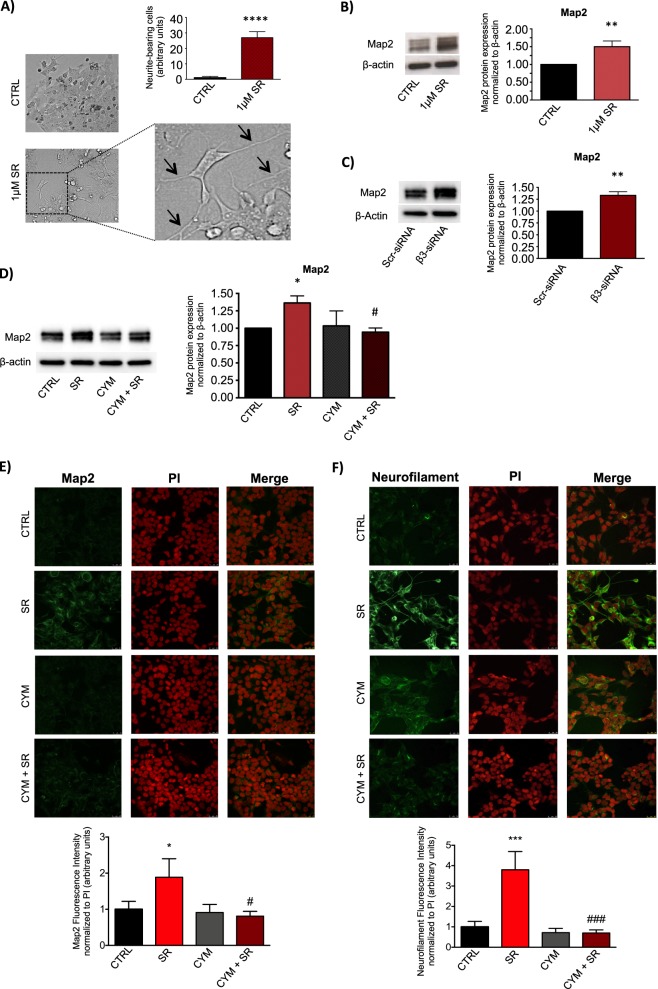
β3-AR blockade increases neuronal differentiation of human neuroblastoma BE(2)C cells through the involvement of SK2/S1P_2_ signaling. **a** Images showing neurite outgrowth (black arrows) and relative quantification in SR59230A and control condition after 72 h of treatment of BE(2)C cells. To quantify neurite outgrowth, images were acquired from six randomly chosen fields in each well in triplicate for condition, and neurite-bearing cells were counted. Results are reported as mean ± SD of three independent experiments. Significance was calculated by Unpaired *t*-test analysis with equal SD (****P < 0.0001). **b** WB and relative densitometric quantification analysis, showing protein levels of the differentiation marker Map2 in BE(2)C cell line treated with 1 μM SR59230A for 48 h. Results were normalized to the expression of β-actin and reported as mean ± SD, fold change over control, set as 1. Blot is representative of three independent experiments. Significance was calculated by Unpaired *t*-test analysis with equal SD (**P < 0.01). **c** WB and relative densitometric quantification analysis, showing protein levels of Map2 in BE(2)C cell line transfected with Scrambled (Scr)- and β3-AR-siRNA for 48 h. Results were normalized to the expression of β-actin and reported as mean ± SD, fold change over control, set as 1. Blot is representative of three independent experiments. Significance was calculated by Unpaired *t*-test analysis with equal SD (**P < 0.01). **d** WB and relative densitometric quantification analysis, showing protein levels of the differentiation marker Map2 in BE(2)C cell line treated with 1 μM SR59230A for 48 h, in presence or not of 10 μM CYM5520. Results were normalized to the expression of β-actin and reported as mean ± SD, fold change over control, set as 1. Blot is representative of three independent experiments. Significance was calculated by two-way ANOVA analysis followed by Bonferroni’s post hoc test (*P < 0.05; ^#^*P* < 0.05 CYM+SR vs. SR). **e** Upper panel: Immunofluorescence images of BE(2)C cells treated with 1 μM SR59230A for 48 h, in presence or not of 10 μM CYM5520 and stained with anti-Map2 antibody, Alexa-fluor488 secondary antibodies and propidium iodide (PI). Images are representative of three independent experiments with similar results. Scale bar 25 µm. Lower panel: Quantification of Map2-associated fluorescence intensity normalized to PI, fold change above control set as 1. Data are mean ± SD of six fields of each specimen quantified in three independent experiments. SR59230A increases Map2 in a statistically significant manner by one-way ANOVA (*P < 0.05); the pharmacological activation of S1P_2_ by CYM5520 abolishes SR59230A-induced increase of Map2 in a statistically significant manner by two-way ANOVA followed by Bonferroni's post hoc test ^*#*^*P* < 0.05. **f** Upper panel: Immunofluorescence images of BE(2)C cells treated with 1 μM SR59230A for 72 h, in presence or not of 10 μM CYM5520 and stained with anti-Neurofilament antibody, Alexa-fluor488 secondary antibodies and PI. Images are representative of three independent experiments with similar results. Scale bar 25 µm. Lower panel: Quantification of Neurofilament-associated fluorescence intensity normalized to PI, fold change above control set as 1. Data are reported as mean ± SD as described above in section e. SR59230A increases Neurofilament in a statistically significant manner by one-way ANOVA (***P < 0.001); the pharmacological activation of S1P_2_ by CYM5520 abolishes SR59230A-induced increase of Neurofilament in a statistically significant way by two-way ANOVA followed by Bonferroni's post hoc test (^###^*P* < 0.001)

Treatment of BE(2)C cells for 72 h with 1 μM SR59230A was able to induce neurite outgrowth (Fig. 4a). Moreover, the neuronal differentiation marker protein Map2 was upregulated at protein level after 24 h of SR59230A treatment (Fig. 4b). To confirm that the SR59230A pharmacologic induction of the neuronal differentiation marker Map2 in NB BE(2)C cells was due to a selective β3-AR-blockade, we performed the silencing of β3-AR mRNA expression. Western blot analysis showed that β3-AR down-regulation switched NB phenotype to neuronal differentiation by increasing the neuronal marker Map2, corroborating the pharmacological approach (Fig. 4c). To investigate the involvement of the S1P_2_ receptor in the neuronal differentiation induced by the β3-AR-blockade, we used the S1P_2_ agonist CYM5520. Interestingly, the upregulation of the differentiation marker Map2 observed after β3-AR-blockade was completely abrogated by treatment with the S1P_2_ receptor agonist CYM5520 (Fig. 4d).

Immunofluorescence staining of Map2 in BE(2)C cells treated with SR59230A, in the presence or absence of the S1P_2_-agonist CYM5520, demonstrated that S1P_2_ is involved in β3-AR downstream effects, since S1P_2_ engagement by the agonist reverted Map2 expression induced by β3-AR blockade (Fig. 4e). In addition, the expression of the late neuronal differentiation marker Neurofilament is in line with previous results, being upregulated when the β3-AR was blocked by SR59230A, whereas it was completely abrogated in the presence of CYM5520 (Fig. 4f).

Our data demonstrate that in NB cancer, β3-AR blockade leads to a switch from a proliferative to a neuronal differentiation state, and that this effect is completely abolished when the S1P_2_ receptor is triggered by a selective agonist. These results prove the existence of a functional cross talk between β3-AR and S1P signaling in regulating the proliferation/differentiation balance in NB cells.

### β3-AR blockade reduces stemness in human NB BE(2)C cells by modulating the SK2/S1P_2_ signaling axis

**Fig. 5:**
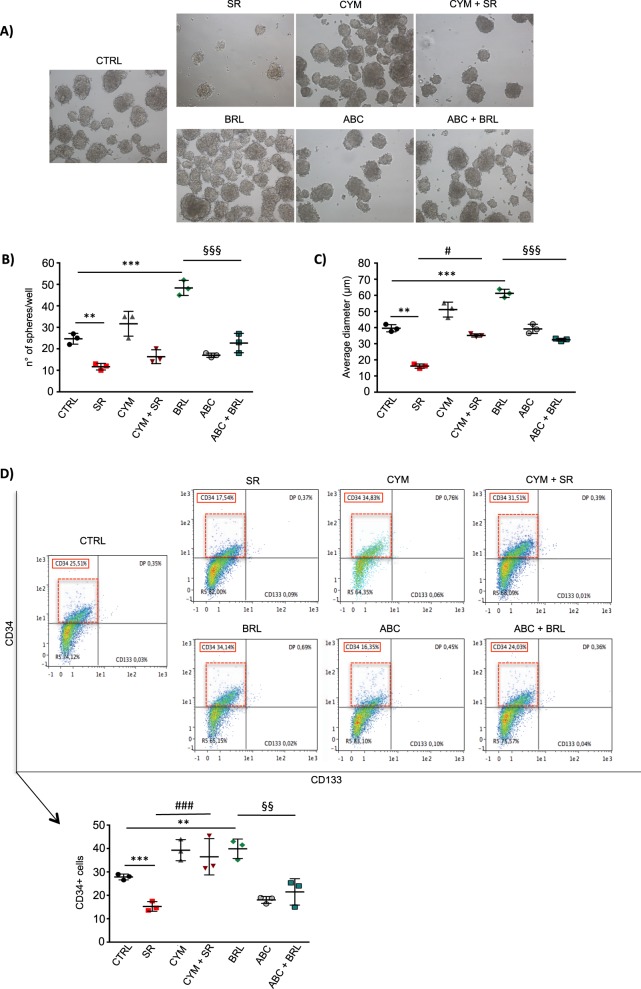
β3-AR blockade reduces stemness in human neuroblastoma BE(2)C cells by modulating the SK2/S1P_2_ signaling axis. Neurospheres formation assay of human neuroblastoma BE(2)C cell line. Cells were plated in MW24 (1 × 10^4^ cells/well) with Neurosphere basic medium (see “Methods” section) and after 24 h treated with 1 μM SR59230A or 1 μM BRL37344, in presence or not of 10 μM CYM5520 or 1 μM ABC294640. Once formed, spheres were disrupted and cells re-plated for a second passage (P2). **a** Images were taken after 7 days of neurosphere formation (P2). The effect of the treatments on stemness features of BE(2)C neurospheres, was evaluated by using different assays. **b** Number of neurospheres formed per well was counted using an optical microscope and compared to the control condition. Results are reported as mean ± SD and are representative of three independent experiments. Significance was calculated by Unpaired *t*-test analysis with equal SD (**P < 0.01, ***P < 0.001) and two-way ANOVA analysis followed by Bonferroni’s post hoc test (^§§§^*P* < 0.001 ABC+BRL vs. BRL). **c** Average diameter of the spheres was quantified by using the ImageJ Software. Results are reported as mean ± SD and are representative of three independent experiments. Significance was calculated by Unpaired *t*-test analysis with equal SD (**P < 0.01, ***P < 0.001) and two-way ANOVA analysis followed by Bonferroni’s post hoc test (^#^*P* < 0.05 CYM+SR vs. SR; ^§§§^*P* < 0.001 ABC+BRL vs. BRL). **d** Flow cytometry analysis of neurospheres was performed by using MACSQuant Analyzer 10 (Miltenyi Biotech). After disruption of the spheres, cells were stained with anti-CD34-PE-Vio770, anti-CD133-APC antibodies and Viobility Fixable dyes. Results were reported as mean ± SD of CD34 positive cells and are representative of three independent experiments. Significance was calculated by Unpaired *t*-test analysis with equal SD (**P < 0.01, ***P < 0.001) and two-way ANOVA analysis followed by Bonferroni’s post hoc test (^###^*P* < 0.001 CYM+SR vs. SR; ^§§^*P* < 0.01 ABC+BRL vs. BRL)

The existence of self-renewing multipotent tumor cells is considered a reservoir of malignant progenitors that are responsible for NB growth and lack of sensitivity to chemotherapeutics [35, 36]. The above-described data on β3-AR role in the differentiation of NB cells, prompted us to investigate whether β3-AR modulation could alter stem cell features in human BE(2)C cells. Neurosphere formation assay was performed and data showed that β3-AR and S1P signaling modulation strongly affected the ability of NB cells to make neurospheres (Fig. 5a). In particular, when β3-AR was blocked by SR59230A the number of neurospheres from BE(2)C cells was significantly decreased, and together with this parameter, neurospheres displayed a smaller diameter, compared to the control condition. Moreover, the administration of S1P_2_ agonist CYM5520 counteracted both these effects brought about by β3-AR-antagonist. On the other side, β3-AR agonist BRL37344 induced a significant increase of stemness potential, quantified by measuring both number and size of neurospheres, and this effect was completely abrogated when the SK2 enzyme was blocked by the selective inhibitor ABC294640 (Fig. 5a–c). Although the expression of the stem cell marker CD133 by flow cytometry analysis of neurospheres showed no appreciable difference when β3-AR and S1P signaling were modulated, the expression of the stem cell marker CD34 was downregulated in the presence of SR59230A, and interestingly, this effect was reverted by CYM5520. Furthermore, BRL37344 increased the CD34 expression in neurospheres, but when SK2 activity was pharmacologically blocked by ABC294640, the expression of the stem marker was similar to the control condition (Fig. 5d), leading to the conclusion that SK2 was involved in the potentiation of stemness downstream of β3-AR in NB.

Taken together these results highlight the role of the cross-talk between β3-AR and SK2/S1P_2_ signaling axis, in controlling the balance between stemness/differentiation of human NB BE(2)C cells.

### β3-AR blockade by SR59230A reduces NB tumor growth in A/J mice, through the involvement of SK2 and S1P_2_

**Fig. 6:**
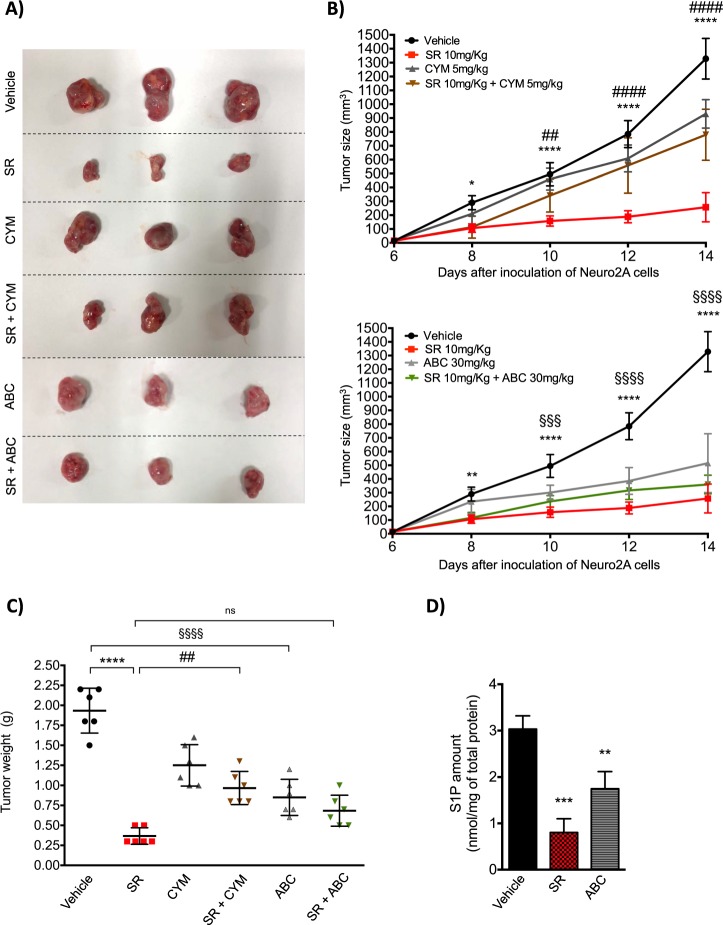
β3-AR blockade reduces tumor growth in A/J mice through the involvement of SK2/S1P_2_ signaling axis. Neuro-2A cells were injected subcutaneously (1 × 10^6^ cells) in the flank of A/J female mice. When a palpable tumor mass was formed (6 days), mice were treated with Vehicle (i.p.), SR59230A 10 mg/kg (i.p.) alone or in combination with CYM5520 5 mg/kg (i.p.) and ABC294640 30 mg/kg (p.o.). **a** After 8 days of treatment, mice were sacrificed and tumor mass excised; images of tumors are representative of the final size of all tumors respectively for each treatment (*n* = 6 for group). **b** Tumor growth rate obtained by measuring tumor size of the mass calculated as Volume = [(length × widht)^2^/2] in Vehicle (*n* = 6), SR59230A (*n* = 6), CYM5520 (*n* = 6) and SR59230A+CYM5520 (*n* = 6) (top) and Vehicle (*n* = 6), SR59230A (*n* = 6), ABC294640 (*n* = 6) and SR59230A+ABC294640 (*n* = 6) (bottom). Vehicle and SR tumor size results reported in (top) and (bottom) graphs are referred to the same tumors. Significance was calculated by two-way ANOVA analysis followed by Bonferroni’s post hoc test (*P < 0.05, **P < 0.01, ****P < 0.0001; ^##^*P* < 0.01 SR+CYM vs. SR, ^####^*P* < 0.0001 SR+CYM vs. SR; ^§§§^*P* < 0.001, ^§§§§^*P* < 0.0001 Vehicle vs. ABC). **c** Tumor weight at the end point of the experiment (14 days) (*n* = 6 per group). Results are reported as individual dots with mean (central line) ±SD. Significance was calculated by two-way ANOVA analysis followed by Bonferroni’s post hoc test (****P < 0.0001; ^##^*P* < 0.01 SR+CYM *vs*. SR; ^§§§§^*P* < 0.0001 ABC vs. Vehicle). **d** S1P measurement in tumors of Vehicle- (*n* = 3), SR59230A- (*n* = 3) and ABC294640-treated mice (*n* = 3). Results are reported as mean ± SD. Significance was calculated by one-way ANOVA analysis followed by Bonferroni’s post hoc test (**P < 0.01, ***P < 0.001)

In different NB cells, several non-selective β-AR antagonists showed reduction of tumor proliferation and viability in vitro and tumor growth in vivo [20, 21]. Nevertheless, the role played by the β3-AR in NB tumor growth has never been investigated. Driven by our in vitro results, we investigated whether the β3-AR antagonist SR59230A could affect tumor growth in a mice syngeneic model of NB cancer. For this purpose, we injected subcutaneously Neuro-2A cells in A/J mice and when tumor mass were palpable, at 6 days, mice were treated every day for 8 days until the sacrifice. As shown in Fig. 6a, b, in mice treated with SR59230A for 8 days, there was a strong reduction of NB tumor growth compared to vehicle-treated mice. In line with previous data obtained in vitro, the tumor growth impairment observed in SR59230A-treated mice was partially reverted by the use of the S1P_2_-agonist CYM5520. Intriguingly, a partial reduction of tumor growth was observed in CYM5520 alone-treated mice that could be explained by an anti-angiogenic action of S1P_2_ receptor as shown in Supplementary Fig. 4. Interestingly, in mice treated with the SK2 inhibitor ABC294640, tumor growth suppression was similar to that observed in SR-treated mice. Moreover, there was no further decrease in tumor size in mice co-treated with the β3-AR antagonist together with SK2 inhibitor, compared to β3-AR antagonist alone. Finally, tumor weight after 8 days of treatments, showed a strong reduction when mice where treated with the β3-AR-antagonist SR59230A. Again, as for the tumor growth rate, this effect was significantly reverted by the S1P_2_-agonist CYM5520, and on the other hand, there was no significant difference when mice where treated with both SR59230A and ABC294640 compared to SR59230A alone (Fig. 6c). The quantification of epinephrine and norepinephrine in plasma of A/J mice treated with SR59230A and ABC294640, in Supplementary Fig. 5a, b, clearly indicated that in our model both SK2 inhibition and β3-AR antagonism were able to decrease plasmatic concentration of norepinephrine, whereas ABC294640 significantly decreased epinephrine amount as well. Moreover, as shown in Fig. 6d, both SR59230A and ABC294640 strongly decreased S1P levels in the tumor mass.

The in vivo results clearly support the involvement of the β3-AR in sustaining the NB tumor progression. Moreover, the specific role played by the β3-AR in NB relies, at least in part, on the S1P signaling pathway via SK2 and S1P_2_ modulation.

### NB tumor growth reduction exerted by SR59230A via modulation of SK2/S1P_2_ signaling axis, is associated to increased neuronal differentiation of NB cells

**Fig. 7:**
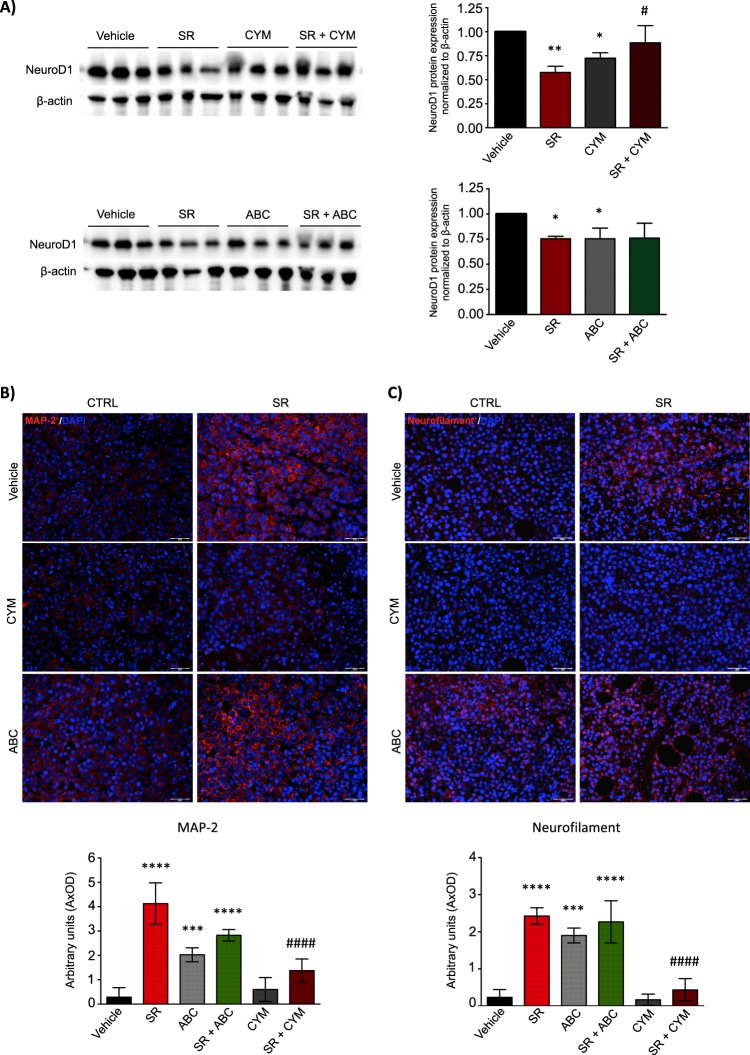
Neuroblastoma tumor growth reduction exerted by SR59230A via SK2/S1P_2_ signaling axis is accompanied with an increased neuronal differentiation of NB cells. **a** WB and relative densitometric quantification analysis, showing the protein expression levels of the early differentiation marker NeuroD1. Lysates were obtained from tumor mass (at 14 days) of Vehicle-, SR59230A-, CYM5520- and SR59230A+CYM5520-treated mice (*n* = 3 for each group) (top) and Vehicle-, SR59230A-, ABC294640- and SR59230A+ABC294640-treated mice (*n* = 3 for each group) (bottom). Significance was calculated by two-way ANOVA analysis followed by Bonferroni’s post hoc test (*P < 0.05, **P < 0.01; ^#^*P* < 0.05 SR+CYM *vs*. SR). **b** Upper panel: Immunofluorescence of paraffin-embedded tumor sections showing expression of the neuronal differentiation marker Map2. Images are representative of similar results obtained for Vehicle- (*n* = 5), SR59230A- (*n* = 5), CYM5520- (*n* = 5), SR59230A+CYM5520- (*n* = 5), ABC294640- (*n* = 5) and SR59230A+ABC294640-treated mice (*n* = 5). Lower panel: Quantification of Map2-associated fluorescence intensity. Data are mean ± SD of eight fields of each specimen quantified in three independent experiments. Significance was calculated by one-way ANOVA analysis followed by Bonferroni’s post hoc test (****P < 0.0001 SR vs. Vehicle, ***P < 0.0001 ABC vs. Vehicle, ****P < 0.0001 SR+ABC vs. Vehicle; ^####^*P* < 0.0001 SR+CYM vs. SR. **c** Upper panel: Immunofluorescence of paraffin-embedded tumor sections showing expression of the neuronal differentiation marker Neurofilament. Images are representative of similar results obtained for Vehicle- (*n* = 5), SR59230A- (*n* = 5), CYM5520- (*n* = 5), SR59230A+CYM5520- (*n* = 5), ABC294640- (*n* = 5) and SR59230A+ABC294640-treated mice (*n* = 5). Lower panel: Quantification of Neurofilament-associated fluorescence intensity. Data are mean ± SD of eight fields of each specimen quantified in three independent experiments. Significance was calculated by one-way ANOVA analysis followed by Bonferroni’s post hoc test (****P < 0.0001 SR vs. Vehicle, ***P < 0.0001 ABC vs. Vehicle, ****P < 0.0001 SR+ABC vs. Vehicle; ^####^*P* < 0.0001 SR+CYM vs. SR

With the specific purpose to analyze the differentiation state in the tumor mass isolated from syngeneic mice treated with β3/SK2/S1P_2_ modulators, we performed WB and IF analyses to study the expression of early, intermediate and late neuronal markers. As shown in Fig. 7a, the β3-AR-antagonist SR59230A and the SK2-inhibitor ABC294640, were both able to decrease the protein expression levels of the early neuronal differentiation marker NeuroD1 in tumor mass excised from A/J syngeneic mice; however, in the presence of the S1P_2_-agonist CYM5520, this effect was abrogated. On the contrary, the expression levels of the intermediate neuronal differentiation marker Map2 and of the late differentiation marker Neurofilament, were upregulated in SR59230A- or ABC294640-treated mice. Strikingly, the expression of these differentiation markers in mice administered with SR59230A in presence of the S1P_2_ agonist CYM5520, were comparable to the vehicle condition (Fig. 7b, c).

The data obtained in vivo corroborate our preliminary results, and highlight the crucial role played by the β3-AR in controlling the tumor growth and the differentiation grade of NB tumor. Moreover, we confirm that the biological effects observed in β3-AR-blockade conditions rely on the modulation of the S1P signaling pathway.

## Discussion

The effects of nonselective β-blocker propranolol in combination with anti-cancer agents has been already reported in different oncologic diseases including NB [21], whereas the specific involvement of β3-AR was not investigated. Recently, β3-AR expression was analyzed in several cancer tissues. Among different tumor specimens, higher levels of β3-AR were detected in melanoma and moderate levels of expression were observed across cancer tissues such as thyroid papillary carcinoma and T-cell lymphoma [17]. Literature data have already shown that β3-AR, which expression increases under hypoxic conditions, is involved in melanoma growth, since its blockade reduced tumor growth in vivo [18]. Moreover, β3-AR activation sustained melanoma growth and aggressiveness by driving stromal microenvironment towards the secretion of angiogenetic and pro-inflammatory cytokines [19], thus involving β3-AR in adaptive responses of melanoma. However, the expression and possible dysregulation of β3-AR have not yet been investigated in NB. Here we show for the first time that β3-AR is constitutively expressed in NB cells, whereas β3-AR is reported to be induced under hypoxia in melanoma cells [18]. In this study, three different NB cell lines have been employed, murine Neuro-2A for the in vivo syngeneic model and human SK-N-BE(2) and BE(2)C for the in vitro experiments, showing for the first time that β3-AR is constitutively expressed in all the above-mentioned NB cells. Noteworthy, higher levels of catecholamine metabolites that characterize NB tumor condition reflect catecholamine production by tumor microenvironment. We can speculate that catecholamines may target cancer cells through the activation of β3-AR, contributing to a feedforward loop of growth and malignancy in NB cancer. Indeed, the decrease of catecholamine levels in plasma from NB mice after β3-AR blockade or SK2 inhibition reinforces our mechanistic model of S1P/β3-AR crosstalk in NB (Supplementary Fig. 5).

We focused our attention to effects elicited by a sublethal dose of β3-AR antagonist SR59230A (1 µM), since its moderate toxicity at concentrations over 5 µM might be partially ascribed to its off-target effect, as we show in non-tumor cell lines such as human fibroblast and endothelial cells (Supplementary Fig. 2). The effects of 1 µM SR59230A treatment as a pharmacological tool to block β3-AR phenocopy those obtained by RNA interference approach, thus confirming in our model the specificity and selectivity of SR59230A for subsequent experiments.

A strong relationship between cellular differentiation and tumorigenesis in NB has come from the Shimada histology-grading system, in which the differentiation grade has an important prognostic significance, being the undifferentiated tumors the most malignant [37]. Besides reducing tumor cell viability, pharmacological tools that hit the stem cell potential and push cells towards differentiation, thus counteracting the onset of chemoresistance, may represent a promising approach to treat NB. In fact, high-risk cases of NB face poor prognosis due to therapy-resistant relapse that relies on the presence of cancer stem cells, characterized by a poorly differentiated phenotype. For this reason, high-risk NB is currently treated with the differentiating agent retinoic acid at completion of cytotoxic therapy. Although this improves survival, in metastatic NB patients the time-free survival rate still remains quite low [38]. Therefore, we focused our attention on neuronal differentiation and performed subsequent experiment by using sublethal doses of SR59230A, dissecting a crucial role for β3-AR in the balance between stemness and differentiation of NB cells. Intriguingly, the analysis of neuronal differentiation of BE(2)C cells by morphological and molecular approaches demonstrated that β3-AR blockade is capable to increase neurite formation and neuronal markers expression. The decreased protein expression of the early neuronal differentiation marker NeuroD1 in NB tumors excised from SR59230A-treated mice, strengthens our in vitro results on the role of the β3-AR in NB biology, being high levels of this marker involved in the promotion of NB progression. Interestingly, NeuroD1 has been also involved in the induction of anaplastic lymphoma kinase (ALK), known to be one of the most important predisposition genes for NB [39] tumorigenesis, thus, hinting that NeuroD1 down-regulation in SR59230A-treated tumors could counteract ALK in sustaining the NB tumorigenesis.

Despite the CD133 antigen has been recognized as one of the most important stemness-related markers in NB [40, 41], we observe that neurospheres from BE(2)C cells show a slight expression of the CD133 antigen, and no significant changed expression of this marker following β3-AR modulation. Instead, the expression of the CD34 antigen is significantly affected by the binding of β3-AR and modulation of S1P signaling. These results are in agreement with literature data, in fact, despite the CD34 antigen was firstly identified as a hematopoietic marker, several studies demonstrated that it represents a specific marker for cancer stem cells in NB. Moreover, NB cells and spheres derived from the bone marrow metastasis of high-risk tumors, as well as our spheres from BE(2)C cell line, do not express appreciable levels of CD133, instead contain CD34 enriched subpopulation, identified as the most tumorigenic cells and responsible for relapse in NB patients [42–45]. Our results therefore reinforce the crucial role played by the β3-AR in controlling the stemness features of tumorigenic subpopulations of cells in NB cancer. Therefore, it can be assumed that catecholamine, besides being released to promote NB growth, modulate the balance between differentiation and stemness of NB cells via β3-AR activation.

Evidence for β3-AR/S1P cross-talk have been recently reported and this molecular interplay seems to be involved in physiopathological effects in different cellular contexts [30, 46]. The occurrence of functional interplay between β3-AR and SK/S1P axis has been demonstrated by both pharmacological and RNA interference approach, and these results were further confirmed by the employment of a specific β3-AR agonist. In our NB cell model, β3-AR antagonist potently down-regulates SK2 and S1P_2_ whereas it does not affect SK1 expression. Conversely, the treatment with BRL37344 inversely affects the expression of SK2 and S1P_2_. We can speculate that an appreciable basal constitutive activity of the β3-AR occurs in NB cells, even though the binding of β3-AR agonist further potentiates receptor activation.

Noteworthy, our results are in agreement with published studies reporting that SK2 is highly expressed in NB, being required for the production of bioactive lipid S1P that via S1P_2_ exerts biological function in NB [27]. Moreover, the sphingosine analogue and pro-drug FTY720 decreases SK2 activation and cellular proliferation of NB in vitro and tumor growth in vivo showing a synergistic effect with topotecan [29].

These results reinforce the notion that SK/S1PR axis is a signaling pathway that can be exploited by different exogenous cues to promote tumor growth and aggressiveness. In particular, the specific cross talk with β3-AR is fundamental for the induction of neuronal differentiation in NB that could be an innovative and efficacious strategy to treat high-risk NB patients. The observation that CYM5520 slightly decreases NB growth in mice can be explained by the negative role of S1P_2_ in tumor angiogenesis (Supplementary Fig. 4), that possibly affects tumor growth in vivo, in agreement with literature data [47]. It is worth to mention that S1P_2_ is widely distributed in tissues and exerts systemic effects that could be difficult to dissect. Anyway, these effects are only apparently in contrast with our mechanistic model, since in the presence of CYM5520 the effect of SR59230A was completely reverted both in vitro and in vivo. We hypothesize that the transactivation of S1P_2_ by β3-AR occurs via the well-known S1P inside-out signaling, that requires the involvement of SK2 activation and S1P release. Indeed, both β3-AR blockade and SK2 inhibition significantly decrease S1P levels in the tumor mass. Although we cannot exclude a partial compensation effect of SK1 after inhibition of SK2 at the level of systemic S1P, whether this occurs it does not revert S1P drop by ABC294640 treatment in tumors.

In summary, our findings highlight the crucial role played by the β3-AR in regulating tumorigenesis of NB. Intensive research aimed at addressing the molecular mechanisms of β3-AR and SK/S1P axis will feasibly identify innovative targets to treat high-risk NB. These findings may have relevant implications for therapeutic purposes, since the available therapies are still not effective enough for metastatic diseases. Furthermore, biologically based treatments that specifically target pathways responsible for malignant transformation and progression in NB could be more effective and less toxic than standard chemotherapeutic agents.

## Materials and methods

### Cell culture

SK-N-BE(2), BE(2)C human NB cancer cells, and Neuro-2A murine NB cancer cells were obtained from ATCC. SK-N-BE(2) and BE(2)C cells were grown in DMEM:F12 supplemented with 10% of FBS, Neuro-2A cells were grown in DMEM supplemented with 10% of FBS. Cell lines were cultured at 37 °C water-satured, 5% CO_2_ atmosphere, and 21% O_2_ for normoxia, and 1% O_2_ for hypoxia.

### Western blot analysis

Cells were collected and lysed in RIPA buffer containing protease inhibitor cocktail. After quantification, 20 μg of total proteins were used to perform an SDS-PAGE and WB analysis. PVDF membranes were incubated overnight with the primary antibodies (anti-Map2, Cell Signaling, #4542; anti-SK1 and anti-SK2, ECM Biosciences, #SP1621 and #SP4621, respectively; anti-S1P_2_, Proteintech, 21180-1-AP; anti-NeuroD1, Abcam, ab60704; anti-β2-AR sc-9042, anti-β3-AR sc-13108 and anti-β-actin sc-1615, Santa Cruz Biotechnology) at 4 °C and then with specific secondary antibodies for 1 h at room temperature. Binding of the antibodies with the specific proteins has been detected by using Clarity Western ECL Substrate (Biorad).

### MTT assay

Viability of tumor cells was evaluated using an MTT assay. NB cells were treated for 24 h with different concentration of SR59230A and then maintained in MTT for 1 h at 37 °C before lysis with an equal volume of DMSO. The absorbance of the solubilized dye was evaluated at 570 nm using a spectrophotometer.

### Real-time PCR

Gene expression analysis was performed using 2^(-ΔΔCT) comparative method of quantification [48]. Briefly, total RNA (1 µg), extracted with TriReagent™ (Sigma-Aldrich S.r.l.) was reverse transcribed using iScript™ cDNA Synthesis Kit for RT-qPCR (Bio-Rad Laboratories S.r.l.) according to the manufacturer's instructions. The quantification of target gene mRNA levels was performed in triplicate for each specimen employing TaqMan Universal Master Mix and the automated ABI Prism 7500 Sequence Detector System (Thermo Fisher Scientific INC). Human specific TaqMan Gene Expression Assays employed for gene expression studies were purchased from Thermo Fisher Scientific INC. Simultaneous amplification of the target sequences (SPHK1:Hs00184211_m1, SPHK2:Hs01016543_g1, SGPL1:Hs00393705_m1, SPNS2:Hs01390449_g1, S1PR1:Hs00173499_m1, S1PR2:Hs01003373_m1, S1PR3:Hs01019574_m1 Gene Expression assays) together with the housekeeping gene, β-actin (ACTB: Hs99999903_m1 Gene Expression assay) was carried out essentially as previously described [49]. Results were analyzed by ABI Prism Sequence Detection Systems software, version 1.7 (Thermo Fisher Scientific INC).

### Cell transfection

Cells grown into tissue culture 6-well plates, and when were 60% confluent were transfected with siRNA duplexes (SASI_Hs01_00015585 and SASI_Hs01_00015586 for β3-AR; SASI_Hs01_00209061 and SASI_Hs01_00209062 for β2-AR) using Lipofectamine RNAiMAX (Thermo Fisher Scientific INC), according to the manufacturer's instructions as previously described [50]. Briefly, Lipofectamine RNAiMAX was incubated with siRNA in DMEM without serum and antibiotics for 20 min, and afterward the lipid/RNA complexes were added to cells to a final concentration of 20 nM in DMEM containing serum. After 24 h, cells were shifted to DMEM without serum and then used for the experiments within 72 h from the beginning of transfection.

### Proliferation assay

Cell proliferation was determined by measuring [^3^H]-thymidine incorporation. BE(2)C cells were serum-starved for 24 h and then challenged with different molecules as described in the figure legend. [^3^H]-thymidine (0.5 μCi/well) was added for the last 4 h of incubation. Cells were washed in ice-cold PBS before 500 μl addition of 10% trichloroacetic acid (TCA) for 5 min at 4 °C. TCA was removed and 250 μl of ethanol:ether (3:1 v/v) solution was added; plates were then collocated under chemical hood until complete evaporation of the solution. Samples were then lysed in 0.25 N NaOH for 2 h at 37 °C. Incorporation of [^3^H]-thymidine was measured by scintillation counting.

### Immunofluorescence of cells and tissues

Immunofluorescence analysis of human and murine tumor mass was performed as follows. Samples were rapidly excised, fixed in buffered 4% formaldehyde for 24 h and paraffin-embedded. Histological sections, 5 µm thick, were cut from samples, were deparaffinised and boiled for 10 min in sodium citrate buffer (10 mM, pH 6.0; Bio-Optica) for antigen retrieval and immunostained over night at 4 °C with rabbit polyclonal anti-Neurofilament antibody (1:100, anti-160kD Neurofilament medium, Abcam, ab64300) and rabbit polyclonal anti-MAP2 antibody (1:50; anti-Map2, Cell Signaling, #4542) for murine tumors, and rabbit polyclonal anti-β3 receptor antibody (1:50, Abcam, ab140713) for human NB. Immune reaction was revealed incubating the section with donkey anti-rabbit Alexa Fluor 594-conjugated IgG (1:350; Jackson Laboratory). After counterstaining with 4,6-diamidino-2-phenylindole (DAPI), representative images were acquired by an Olympus BX63 microscope coupled to CellSens Dimension Imaging Software version 1.6 (Olympus).

Immunofluorescence analysis of BE(2)C cells was performed as follows. BE(2)C were seeded on microscope slides poly-lysine coated (35,000 cells/well in Nunc Lab-Tek chamber slide 4) and at 60–70% confluence were serum-starved, then pre-incubated with pharmacological drugs 30 min before being challenged with 1 μM SR59230A at 37 °C. Immunofluorescence analysis was performed essentially as previously described [50]. Briefly, after different time intervals indicated in legends, cells were fixed in 2% paraformaldehyde in PBS for 20 min. Permeabilization and quenching were obtained by adding a solution containing Triton X-100 0.1% and ethanolamine (1: 165) for 30 min at room temperature. Cells were then blocked in 1% BSA for 1 h and incubated with primary antibodies (anti-160kD Neurofilament medium, Abcam, ab64300; anti-Map2, Cell Signaling, #4542) for 1 h and subsequently, after repeated washes in PBS, slides were incubated with Alexa-fluor488 secondary antibodies. Images were obtained using a Leica SP8 laser scanning confocal microscope (Leica Microsystems GmbH, Wetzlar, Germany) with a 63X objective.

### Neurosphere assay

For neurosphere formation assay, 24-well plates were coated with 1,2% of Poly(2-hydroxyethyl methacrylate) diluted in 95% ethanol. Then, cells were plated (5.000/well) in Neurosphere basic medium composed of DMEM:F12 supplemented with 2% B27, 1% N2, 20 ng/ml FGF and 20 ng/ml EGF. After 24 h, cells were treated with 1 μM SR59230A and 1 μM BRL37344 alone, or in combination with 1 μM ABC294640 and 10 μM CYM5520. Once formed, spheres were disrupted and cells re-plated for a second passage (P2). After 7 days, neurosphere were counted and the diameter size measured using the ImageJ software (National Institutes of Health, U.S.). Neurosphere were then disrupted and stained for a flow cytometry analysis.

### Flow cytometry analysis

To evaluate the expression of the antigens CD34 and CD133, neurospheres of human NB BE(2)C cells (P2) were mechanically dissociated, and single cells suspended in staining buffer. Cells were than treated with FcR blocking reagent and then stained with Viobility 488/520 Fixable dye, anti-CD34(PE-Vio770) and anti-CD-133(APC) antibodies (Miltenyi Biotec). After staining, cells were subjected to flow cytometry by using a Miltenyi Biotec MACSQuant Analyzer 10. Results were analyzed by using Flowlogic^TM^ Software.

### S1P quantification

The S1P concentration in tumor mass was determined using the Sphingosine 1-phosphate Assay Kit (Echelon Biosciences, K-1900) according to the manufacturer’s instructions. The absorbance at 450 nm was measured, and the concentration of S1P in the samples was determined by comparison with the standard curve.

### Tumor syngeneic model

Female NCI A/JCr mice 4-weeks-old were bought from Charles River Laboratories (Frederick). Neuro-2A cells were subcutaneously implanted in A/J recipient mice by injecting 1 × 10^6^ cells in 100 µl of PBS in the right flank. When Neuro-2A cells formed a palpable tumor (about 6 days), treatments started. The treatments were administrated twice a day for SR59230A and Vehicle, and once a day for ABC294640 and CYM5520. SR59230A (Tocris Bioscence) was delivered at 10 mg/kg of physiological solution via intraperitoneal (i.p.); ABC294640 (MedChem Express) was delivered at 30 mg/kg in 0,375% of Polysorbate 80 in PBS via per os (p.o); CYM5520 (Sigma Aldrich) was delivered at 5 mg/kg in 3.6% DMSO in PBS via i.p. Tumor growth rate was evaluated by measuring tumor mass with a caliber, and tumor mass volume calculated as Volume = [(length × width)^2^/2]. Mice were sacrificed after 8 days of treatment.

### Statistics

Densitometric analysis of the WB bands was performed by ImageJ software. Graphical representations were obtained by GraphPad Prism 6.0 (GraphPad Software, San Diego, CA), and statistical analyses were performed using Unpaired student’s *t*-test, one-way and two-way ANOVA analysis of variance followed by Bonferroni’s post hoc test. For MTT survival assay significance was calculated by one-way ANOVA analysis followed by Bonferroni’s post hoc test. For Real Time-PCR significance was calculated by Unpaired *t*-test analysis with equal SD. For [^3^H]-thymidine Proliferation Assay significance was calculated by Unpaired *t*-test analysis with equal SD. For the analysis of neurosphere significance was calculated by Unpaired *t*-test analysis with equal SD and two-way ANOVA analysis followed by Bonferroni’s post hoc test. For tumor growth rate significance was calculated by two-way ANOVA analysis followed by Bonferroni’s post hoc test. For the analysis of tumors weight significance was calculated by two-way ANOVA analysis followed by Bonferroni’s post hoc test. Asterisks show statistical significance as reported in figure legends.

## Supplementary information

Supplementary Figure legends and methods

Supplementary - Figure 1

Supplementary - Figure 2

Supplementary - Figure 3

Supplementary - Figure 4

Supplementary - Figure 5
