# Supplementary material for: β3-adrenoreceptor blockade reduces tumor growth and increases neuronal differentiation in neuroblastoma via SK2/S1P2 modulation
Source: Oncogene. 2019 Sep 2;39(2):368–84. doi: 10.1038/s41388-019-0993-1 (PMC6949192; doi:10.1038/s41388-019-0993-1)
Supplement: Supplementary file 1 — Supplementary Figure legends and methods [file 41388_2019_993_MOESM1_ESM.docx]

**Supplementary Figure legends**

**Supplementary Figure 1.** Immunofluorescence staining of β3-AR on pulmonary bronchus (left) as positive control (ref. Bossard F, Silantieff E, Lavazais-Blancou E, Robay A, Sagan C, Rozec B and Gauthier C. β1, β2, and β3 Adrenoceptors and Na+/H+ Exchanger Regulatory Factor 1 Expression in Human Bronchi and Their Modifications in Cystic Fibrosis. *Am J Respir Cell Mol* *Biol*, 2011; **44**:91–98), and epidermis (right) as negative control (ref. Human Protein Atlas: <https://www.proteinatlas.org/ENSG00000188778-ADRB3/tissue/skin>) of β3-AR antibody used for staining of NB tumor sections in Figure 1B.

**Supplementary Figure 2.** MTT survival assay in human endothelial cells HMEC-1, and human fibroblast IMR-90, treated with different concentration of SR59230A for 24 hours. Results are reported as mean ± SD of three independent experiments performed in triplicate. Significance was calculated by one-way ANOVA analysis followed by Bonferroni’s post-hoc test (* *P* < 0.05, ***P* < 0.01, *****P* < 0.0001).

**Supplementary Figure 3.** WB and relative densitometric quantification analysis, showing protein expression levels of SK2, S1P_2_ and MAP2 in NB BE(2)C cell line after 24 hours of 1μM BRL37344 treatment. Results were normalized to the expression of β-actin and reported as mean ± SD, fold change over control, set as 1. Blots are representative of three independent experiments. Significance was calculated by Unpaired t-test analysis with equal SD (***P* < 0.01, ****P* < 0.001).

**Supplementary Figure 4.** Immunofluorescence of paraffin embedded tumor sections showing expression of the endothelial marker CD31, and relative immunofluorescence quantification. Images are representative of similar results obtained for Vehicle- (n=3), CYM5520- (n=3). Significance was calculated by Unpaired t-test analysis with equal SD (****P* < 0.001).

**Supplementary Figure 5. (A)** Epinephrine and **(B)** norepinephrine measurement in plasma samples of Vehicle- (n=3), SR59230A- (n=3) and ABC294640-treated mice (n=3). Results are reported as mean ± SD. Significance was calculated by one-way ANOVA analysis followed by Bonferroni’s post-hoc test (* *P* < 0.05, ***P* < 0.01).

**Supplementary Methods**

**Immunofluorescence of human tissues and murine tumors**

Immunofluorescence analysis of human tissues and murine tumor mass were performed as described in Materials and Methods section (Immunofluorescence of cells and tissues). For β3-AR staining, an anti-β3-AR antibody, Abcam - ab140713 was used (Supplementary – Figure 1). For CD-31 staining in tumor mass (Supplementary – Figure 4), an anti-CD31 antibody, Abcam - ab9498 was used.

**Catecholamine measurement**

Epinephrine and norepinephrine quantification was performed in plasma of mice using the CatCombi ELISA (IBL International GmbH) Assay kit, according to the manufacturer’s instructions. The absorbance at 405 nm was measured, and the concentration of Adrenalin and Noradrenalin in the samples was determined by comparison with the standard curve.
