# Supplementary figures and images for: β3-adrenoreceptor blockade reduces tumor growth and increases neuronal differentiation in neuroblastoma via SK2/S1P2 modulation
Source: Oncogene. 2019 Sep 2;39(2):368–84. doi: 10.1038/s41388-019-0993-1 (PMC6949192; doi:10.1038/s41388-019-0993-1)

## Supplementary – Figure 1

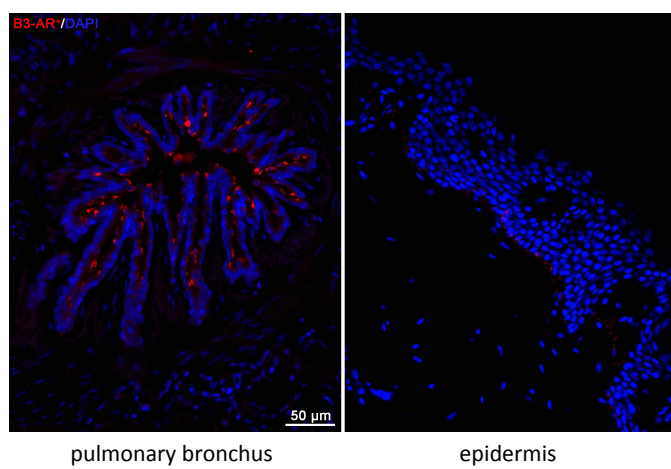

Supplement: Supplementary file 2 — Supplementary - Figure 1 [file 41388_2019_993_MOESM2_ESM.pdf]

Supplementary – Figure 2

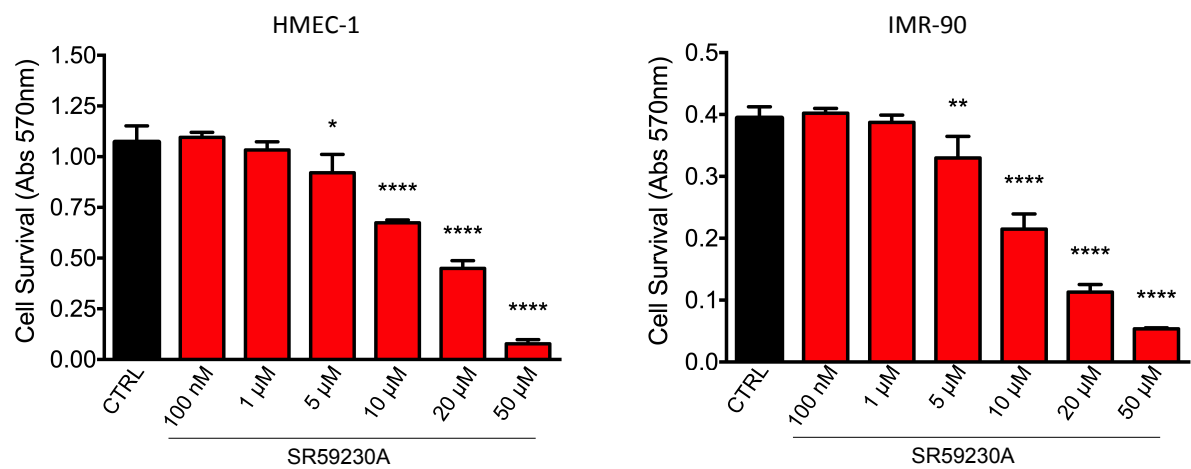

Supplement: Supplementary file 3 — Supplementary - Figure 2 [file 41388_2019_993_MOESM3_ESM.pdf]

Supplementary – Figure 3

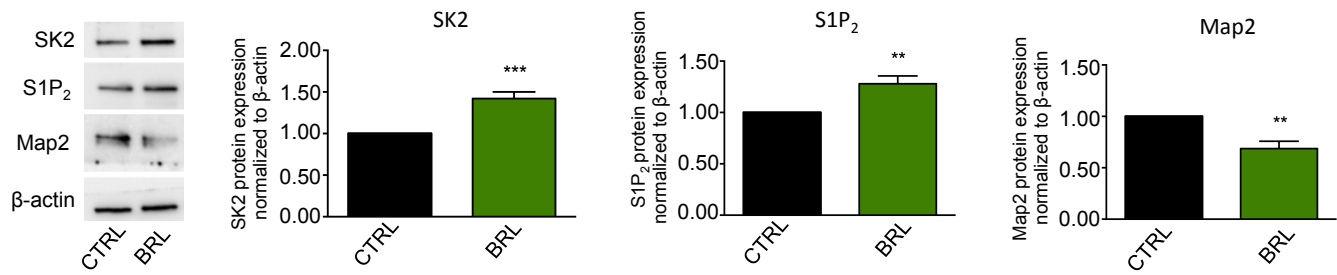

Supplement: Supplementary file 4 — Supplementary - Figure 3 [file 41388_2019_993_MOESM4_ESM.pdf]

## Supplementary – Figure 4

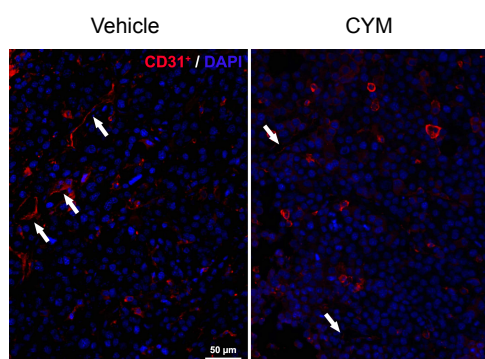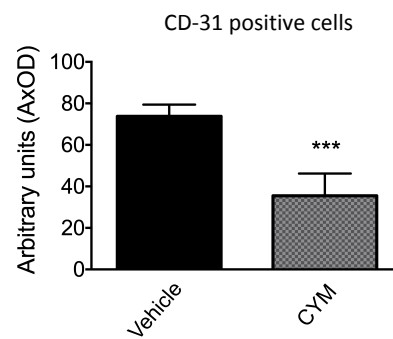

Supplement: Supplementary file 5 — Supplementary - Figure 4 [file 41388_2019_993_MOESM5_ESM.pdf]

Supplementary – Figure 5

A)

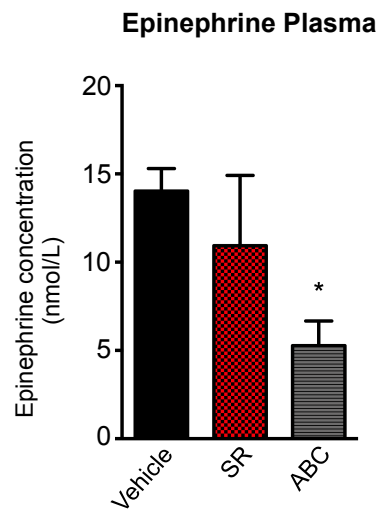

B)

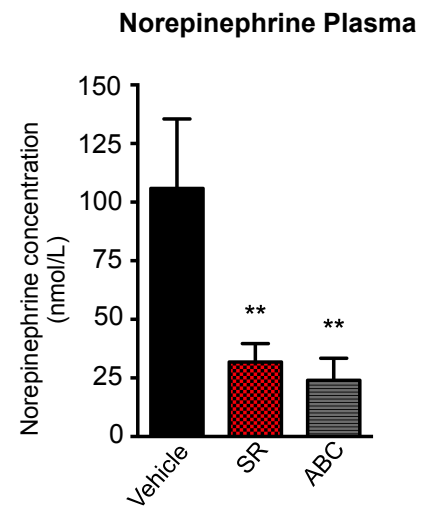

Supplement: Supplementary file 6 — Supplementary - Figure 5 [file 41388_2019_993_MOESM6_ESM.pdf]
